# Plant Virus Biodiversity and Ecology

**DOI:** 10.1371/journal.pbio.0040080

**Published:** 2006-03-14

**Authors:** Jonathan D Wren, Marilyn J Roossinck, Richard S Nelson, Kay Scheets, Michael W Palmer, Ulrich Melcher

## Abstract

The Plant Virus Biodiversity and Ecology (PVBE) project has been initiated to survey the biodiversity of viruses affecting vascular plants.

The International Committee for the Taxonomy of Viruses recognized a global total of about 2,000 species of viruses as of 2005 [1]. However, data on viruses that infect terrestrial organisms, including plants, are sparse, and recent sequencing of samples from marine environments for viruses suggests that 2,000 is a gross underestimate of the total number of viral species on earth [2]. In addition to the paucity of data on viral diversity, little is known about the level of mixed virus infections or temporal patterns of virus accumulation in native plants. For both social and scientific reasons, it is important to extend our knowledge of viruses and their hosts beyond those combinations currently known to cause disease to those that may cause disease in the future (i.e., emerging threats). In addition, we need to gain a greater understanding of the potential for mutualistic interactions between viruses and their hosts. That is, we traditionally view viruses as parasites, which is not surprising given their prominent role as pathogens, but this is not always the case. Consider, for example, polydnavirus–braconid wasp interactions, in which the virus carries the essential genes required to suppress the immune system of the lepidopteran hosts of the wasp [3]. This is a frontier that is largely unexplored. The Plant Virus Biodiversity and Ecology (PVBE) project has been initiated to survey the biodiversity of viruses affecting vascular plants, including their endophytic fungi, in The Nature Conservancy's Tallgrass Prairie Preserve of Oklahoma, home to over 700 plant species. Plants are an ideal starting point for studies on virus ecology since they are immobile and can be readily resampled. Viral screening is done using double-stranded RNA analysis, cloning, sequencing, and microarray analysis. The PVBE effort is not directed toward studying economically important or symptomatic plants, both of which are already heavily studied. Information in Figure 1, for example, summarizes the initial plant source of viruses cataloged in the Viral Identification Data Exchange database [4]. Most of the available virus information we have is derived from cultivated crop species, yet these species only comprise a minute fraction of all plant species. And much of this information is derived from symptomatic hosts, yet only a small fraction of viruses probably cause disease [5]. Furthermore, much of the data gathered on viruses come from monocultures, which favors a low diversity. Thus, the data gathered so far have not only been heavily skewed toward crop-affecting viruses, but also toward standard, laboratory-strain, phenotype-producing viruses. The presence of viruses in plants seems to be relatively common—for example, approximately 60% of plants surveyed in a Costa Rican region containing about 7,000 plant species total were positive for double-stranded RNA, a marker suggesting the presence of viruses (M. J. Roossinck, unpublished data). Taken together, these observations suggest a huge gap in our overall understanding of viral diversity, evolution, and ecology.

**Figure 1:**
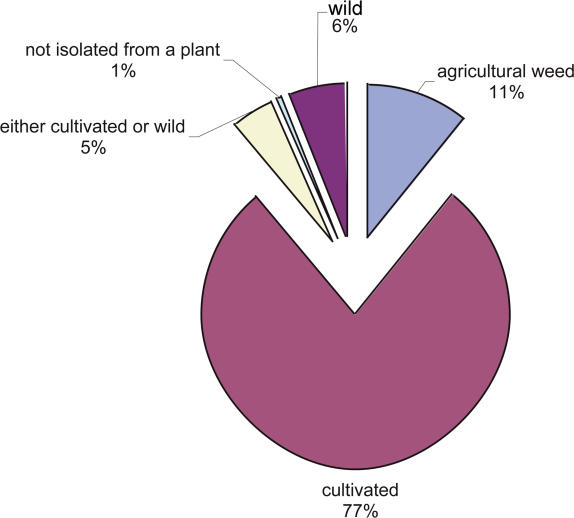
Frequencies of Sources of Plants from Which Recognized Plant Viruses Were Initially Isolated

Closing this gap begins with a broad cataloging and study of viruses affecting wild plants, whether symptomatic or asymptomatic, to gain a more objective view of virus populations in nature. Ostensibly, it ends with a better understanding of the mutualistic interactions between viruses and their hosts. Newly discovered viruses will provide information for those doing basic research on the mechanisms of virus replication, translation, particle assembly, and movement, as well as provide tools for biotechnology (e.g., potential new vectors for gene knockdown studies through virus-induced gene silencing). It is possible that some of these new viruses may later be recognized as the origins of future crop scourges. Knowledge now of their existence and ecology will allow us to better prepare and prevent the catastrophic outcomes often associated with such outbreaks. Gathering information on these viruses will also increase our ability to do more extensive sequence comparisons between viruses, thereby allowing better predictions for active site identities within viral proteins, and to identify novel viral proteins and their functions.

Not only will these efforts help us understand virus ecology, but there is also great potential for revolutionizing plant ecology. The extended phenotype of a virus may affect a plant's local adaptation to its environment. Endophytes, mycorrhizae, or other symbionts could potentially mediate such interactions. Given that many plant viruses are generalists with respect to host species, it is theoretically possible that such effects may be ecosystem-wide. RNA silencing [6] has dramatic but unexplored ecological implications. As a hypothetical example, a bison-borne virus could silence genes for antigrazing defenses, thus facilitating its transmission.

Known virus–vector–host systems exhibit a bewildering complexity. It is increasingly evident that the simple view of viruses as pathogens is outdated. As “little pieces of genetic information” transferred from one organism to another, they have potential for facilitating all sorts of interactions among macroscopic life. Given intimate genetic interactions with their hosts, viruses could potentially be prime drivers of evolutionary change. The degree to which viruses have determined micro- and macro-evolutionary patterns in vascular plants is mostly unexplored, and the relevant questions are not obvious. Comparing virus phylogenies to plant phylogenies will allow us to generate hypotheses about the nature of their coevolution and to push ecology beyond the simple concepts of competition, mutualism, parasitism, and predation.

The PVBE effort faces multiple challenges, including creating resources for an effort sustainable beyond the funding period and providing tools for the analysis of geographic and temporal infection patterns that can be applied to other systems. One of our goals is to establish a community resource for information on plant viruses and their relationships with noncrop plant hosts. This will help us to better estimate the global number of plant viruses and to address the following important scientific questions: what is the relationship between virus biodiversity and host biodiversity? how often do coinfections by different viruses occur in natural systems? and what is the incidence of disease-inducing viruses compared with asymptomatic or beneficial viruses in natural systems? We anticipate a wealth of data from The Nature Conservancy's Tallgrass Prairie Preserve area alone, but the addition of other geographical areas will eventually provide a much bigger picture of continental and global trends in plant virus biodiversity and ecology, a new and emerging field of study that will hopefully enable us to answer these important questions.

## Abbreviation

PVBE: Plant Virus Biodiversity and Ecology
